# In Uveal Melanoma, Angiopoietin-2 but Not Angiopoietin-1 Is Increased in High-Risk Tumors, Providing a Potential Druggable Target

**DOI:** 10.3390/cancers13163986

**Published:** 2021-08-07

**Authors:** Anna M.W. ten Voorde, Annemijn P.A. Wierenga, Rogier J. Nell, Pieter A. van der Velden, Gregorius P.M. Luyten, Robert M. Verdijk, Martine J. Jager

**Affiliations:** 1 Department of Ophthalmology, Leiden University Medical Center, 2333 ZA Leiden, The Netherlands; a.m.w.ten_voorde@lumc.nl (A.M.W.t.V.); a.p.a.wierenga@lumc.nl (A.P.A.W.); R.J.Nell@lumc.nl (R.J.N.); p.a.van_der_velden@lumc.nl (P.A.v.d.V.); g.p.m.luyten@lumc.nl (G.P.M.L.); 2 Department of Pathology, Leiden University Medical Center, 2333 ZA Leiden, The Netherlands; r.m.verdijk@lumc.nl; 3 Department of Pathology, Section Ophthalmic Pathology, Erasmus MC, University Medical Center Rotterdam, 3015 GD Rotterdam, The Netherlands

**Keywords:** eye disease, uveal melanoma, inflammation, oncology, angiogenesis

## Abstract

**Simple Summary:**

We hypothesize that proangiogenic factors such as angiopoietin-1 (ANG-1) and angiopoietin-2 (ANG-2), two targetable cytokines, may play a role in tumor development in uveal melanoma. We determined the expression of these cytokines in both uveal melanoma tissue as well as in aqueous humor, which provides a unique combination of data. We observed that ANG-2, in contrast to ANG-1, showed more expression in high-risk tumors and was associated with the development of metastases. Its presence in aqueous humor correlated with expression in tumor tissue. Knowledge about the expression of these cytokines may help to identify targets for personalized treatment.

**Abstract:**

Uveal melanoma (UM) metastasize haematogeneously, and tumor blood vessel density is an important prognostic factor. We hypothesized that proangiogenic factors such as angiopoietin-1 (ANG-1) and angiopoietin-2 (ANG-2), two targetable cytokines, might play a role in tumor development and metastatic behavior. mRNA levels of ANG-1 and ANG-2 were determined in 64 tumors using an Illumina HT-12 v4 mRNA chip and compared to clinical, pathologic, and genetic tumor parameters. Tissue expression was also determined by immunohistochemistry (IHC). Samples of aqueous humor were collected from 83 UM-containing enucleated eyes and protein levels that were determined in a multiplex proximity extension assay. High tissue gene expression of ANG-2, but not of ANG-1, was associated with high tumor thickness, high largest basal diameter, involvement of the ciliary body, and with UM-related death (ANG-2 mRNA *p* < 0.001; ANG-2 aqueous protein *p* < 0.001). The presence of the ANG-2 protein in aqueous humor correlated with its mRNA expression in the tumor (r = 0.309, *p* = 0.03). IHC showed that ANG-2 was expressed in macrophages as well as tumor cells. The presence of ANG-2 in the tumor and in aqueous humor, especially in high-risk tumors, make ANG-2 a potential targetable cytokine in uveal melanoma.

## 1. Introduction

Uveal melanoma (UM) is a rare type of cancer, but it is the most common primary tumor of the eye, with an estimated incidence rate of 5–9 per 1.000.000 person–years [1]. Despite successful treatment of the primary tumor, about 50% of all patients develop metastases [2,3]. Metastases can occur even up to 15 years after the initial diagnosis, suggesting the existence of micro-metastases at the time of primary treatment [2,4]. Once metastatic disease has developed, survival is short, with a median survival rate of only 6 months [2,5]. Because of this poor prognosis and lack of improvement over time, it is of great importance to identify novel targets for the effective treatment of metastatic UM or for the prevention of metastatic outgrowth.

As UM metastasizes only through hematogenous dissemination [6], the presence of blood vessels is crucial for UM to progress and metastasize [7]. Two recent studies from our laboratory demonstrated that tumor angiogenesis is related to the genetic profile of prognostically bad UM [8,9]. The identification of relevant proangiogenic factors may provide an opportunity to develop new therapeutic targets. We already know that several proangiogenic factors are associated with an increased malignancy of UM, such as vascular endothelial growth factor A (VEGF-A) and hepatocyte growth factor (HGF) [10,11,12,13]. Tumor-infiltrating leukocytes may be a source of proangiogenic cytokines, and, in UM, the presence of an inflammatory phenotype is associated with a bad prognosis. This inflammatory phenotype is characterized by an increased expression of human leukocyte antigens (HLA) class I and II, and infiltration with both lymphocytes as well as macrophages [14,15,16,17]. The main type of infiltrating macrophage was found to be the M2 macrophage, which has angiogenic and tumor-promoting capacities; in UM, a high density of M2 macrophages has been associated with an increased vessel density and a bad prognosis [18,19,20].

Angiopoietin-1 (ANG-1) and angiopoietin-2 (ANG-2) are proangiogenic factors that exert a crucial role in the angiogenic switch, which is associated with tumor progression through interaction with the transmembrane tyrosine protein kinase angiopoietin (Tie2) receptor. This Tie2 receptor is expressed by endothelial cells and tumor-associated macrophages (TAMs) [21,22]. ANG-1 plays an essential role in vessel maturation and regulates the migration, adhesion, and survival of endothelial cells. ANG-2, which is a competitive antagonist of ANG-1, reduces the stabilization of vessels by stimulating the dissociation of pericytes from pre-existing vessels. This leads to an increased vascular permeability, which facilitates the infiltration of cytokines, proteases, and proangiogenic cells [21,23]. Following the example of VEGF, studies have analyzed the effects of monoclonal antibodies directed against these molecules. In a recent study in an experimental colon carcinoma model, tumor-bearing mice received irradiation together with ANG-2-blocking antibodies [24]. The addition of anti-ANG2 antibodies to whole body irradiation improved tumor inhibition, reduced blood vessel density, and led to higher numbers of infiltrating T cells and monocytes than after irradiation alone. A combined treatment with a T cell enhancer, anti-CD40, further promoted the effect of the anti-vascular antibodies, and led to infiltration with CD8+ T cells, considered important for tumor cell killing [25].

Various studies have investigated the function of ANG-1 and ANG-2 in tumor growth and in eye diseases [26,27]. Experimental upregulation of ANG-1 expression provokes tumor growth in various tumor models [27,28,29]. However, an evident correlation between ANG-1 expression and tumor malignancy has never been found [27]. ANG-2 expression is upregulated in tumor-associated vessel endothelium, and high serum ANG-2 concentrations are associated with increased malignancy in several types of cancer, such as hepatocellular cancer, lung cancer, glioblastoma, and cutaneous melanoma [30,31,32,33,34,35,36]. Moreover, an induced expression of ANG-2 has been described as promoting the development of metastases, while specific blockade of ANG-2 inhibited metastasis formation in tumor models in mice [37]. One study investigated the role of ANG-2 in ocular angiogenesis: expression levels of ANG-2 were much higher in the vitreous fluid of eyes with proliferative diabetic retinopathy (PDR), suggesting an association between ANG-2 and retinal angiogenic activity [38]. A recent study in UM from our own group showed that an increased mRNA expression of ANG-2 is related to an increased vessel density and genetic tumor progression [8]. Combining angiogenesis inhibitors and immune checkpoint inhibitors or immune stimulators such as CD40 might be an option for the treatment of UM metastases. The angiopoietin/Tie2 pathway may similarly be an attractive target to prevent metastases. The aim of the current study was to analyze ANG-1 and ANG-2 protein and gene expression in UM tissue and aqueous humor to gain an insight into the potential value of these proteins as therapeutic targets in UM. Furthermore, we determined whether aqueous humor sampling could potentially provide a method to identify which UM patients could be candidates for treatment.

## 2. Materials and Methods

### 2.1. Study Population

Tumor tissue samples were obtained from 64 eyes and samples of aqueous humor from 83 eyes that had undergone primary enucleation for UM between 1999 and 2017 at the Leiden University Medical Center (Leiden, The Netherlands). Tumor tissues and aqueous humor were made available for medical research following pathological evaluation of the tumor-containing eye. The clinical data and survival information of all patients participating in this retrospective cohort study were updated in November 2019. All medical records were analyzed for clinical information (age at enucleation, development of metastases) and pathology reports were used to obtain histopathological characteristics, including tumor size (including both largest basal diameter and prominence), ciliary body involvement, cell type, tumor node metastasis (TNM)/American Joint Committee of Cancer (AJCC) Edition 8 stage and chromosome status [39]. Clinical and histopathologic characteristics of the group are shown in Table 1 and Table 2.

The follow-up time was either the time from enucleation until date of death, or until the last date of follow-up, in cases where the patient was still alive. The mean follow-up period at the time of statistical analysis was 71 months (median, 52, range 2–230 months). During the follow-up period, 46 of the 64 patients of the mRNA study and 57 of the 83 patients in the aqueous humor cohort died, of whom 33 and 40, respectively, died due to metastatic UM. No patients were lost to follow-up.

### 2.2. Immunohistochemical Staining

Immunohistochemical staining (IHC) was performed using the ANG-2 (F-1) antibody from Santa Cruz Biotechnology (Product# SC-74403 via Bio-connect, life sciences, Huissen, The Netherlands). For CD3 staining, we used as primary antibody rabbit monoclonal anti-CD3 (clone: 2GV6, catalog#: 790-4341, Ventana Medical Systems, Inc., Ventana, Rotkreuz, Switzerland). For staining of CD163, the primary antibody of Roche tissue diagnostics (clone: MRQ-26) was used (catalog# 760-4437, Roche diagnostics, Rotkreuz, Switzerland). The UltraView Universal Alkaline Phosphatase Red detection kit (Ventana Ref 760-501, Arizona, United States) was used in combination with target amplification (Ventana Ref 760-080, Arizona, United States). The slides were counterstained with haematoxylin II for 8 min, followed by an additional counterstaining with bluing reagent (Ventana Medical Systems, Inc., Arizona, United States).

### 2.3. Gene Expression Analysis

mRNA for gene expression profiling was obtained from 64 tumors, using the RNA-easy mini kit (Qiagen, Venlo, The Netherlands). ANG-1 and ANG-2 mRNA expression was determined with the Illumina HumanHT-12 v4 chip (Illumina, San Diego, CA, USA), using probes ILMN_1677723 and ILMN_3250067, respectively. For the correlation analysis with proinflammatory proteins, we determined the mRNA expression of CD3 and CD8 lymphocytes, as well as of CD68 macrophages, using probes ILMN_2261416, ILMN_2353732, and ILMN_1714861, respectively, since these probes were found to be the most accurate based on prior validation studies (GEO dataset GSE84976) [40].

### 2.4. Protein Expression Analysis in Aqueous Humor

Protein expression of ANG-1 and ANG-2 in aqueous humor was analyzed using a multiplex proximity extension assay, as previously described (Olink Bioscience, Uppsala, Sweden) [41,42]. During the process, 1 µL of each sample of aqueous humor was incubated with corresponding antibody pairs. Each antibody was linked to a DNA-tag of which the DNA-tags for an antibody pair were complementary. Once the two antibodies were bound to the same and correct target protein, the DNA-tags hybridized and created a double-stranded DNA template. This DNA template was thereafter amplified using a regular PCR. Subsequently, real time PCR was used for quantification of the DNA barcodes (BioMark™ HD System, Fluidigm Corporation, South San Francisco, CA, USA). The generated signal directly corresponded with protein concentration.

Protein expression data were provided as normalized data to minimize the variation within and between different runs as normalized protein expression (NPX). A difference of one in NPX corresponds to a two-fold difference in protein concentration.

### 2.5. Statistical Analysis

All statistical analyses were performed with the statistical software program SPSS (SPSS Inc., Chicago, IL, USA). The protein and gene expression of ANG-1 and ANG-2 in aqueous humor and tumor material were dichotomized into low and high by using the median as cut-off. All clinical, histopathological, and genetic parameters were compared between both groups. For all the categorical prognostic parameters, the Pearson’s chi-squared test was performed and, to compare the continuous prognostic parameters, the Mann–Whitney U test was used. Kaplan–Meier survival analyses, combined with the log-rank test, were performed to analyze disease-specific survival. In these analyses, death due to metastatic UM was considered an event. Apart from the Kaplan–Meier analyses, we performed a univariate survival analysis using a Cox proportional hazard model to identify individual prognostic factors. Subsequently, we performed a multivariate Cox regression analysis to determine independent prognostic parameters. Correlation analyses were performed with Spearman’s rank order correlation. For all statistical tests, a *p*-value < 0.05 was considered statistically significant.

## 3. Results

### 3.1. Distribution of ANG-1 and ANG-2 Gene Expression in UM Tissue

Our lab previously reported a correlation between a high microvascular density and a high expression of ANG-2 in a set of 64 UM tissues [8]. Such a correlation suggests that angiopoietins may be treatment targets in primary and metastatic UM. We used the ANG-1 and ANG-2 gene expression data from the same 64-case cohort to compare expression with clinical, histological, and genetic tumor characteristics [8]. For this, we compared tumors with a low and high gene expression of ANG-1 and ANG-2, split at the median. The median gene expression of ANG-1 was 6.57 Illumina units (mean 6.59, range 6.23–7.04), and the median gene expression of ANG-2 was 6.46 Illumina units (mean 6.53, range 6.12–8.19). Compared to tumors with a low ANG-1 expression (Table 1), the tumors in the high ANG-1 expression group more often showed ciliary body involvement (51% versus 19%, *p* = 0.002), and AJCC-stage III (54% versus 17%, *p* = 0.001), while there was no relation with metastasis formation. With regard to ANG-2 expression, a high gene expression was related to the presence of several high-risk factors: a comparison of the high ANG-2 group versus the low ANG-2 group showed significant differences with regard to age (69.1 versus 56.4 years, *p* = 0.04), largest basal diameter (15 versus 12 mm, *p* = 0.01), mixed or epithelioid cell type (78% versus 53%, *p* = 0.04), AJCC-stage III (50% versus 22%, *p* = 0.02), the presence of monosomy 3 (81% versus 47%, *p* = 0.004), and the occurrence of metastases (78% versus 38%, *p* = 0.001). 

**Figure 1:**
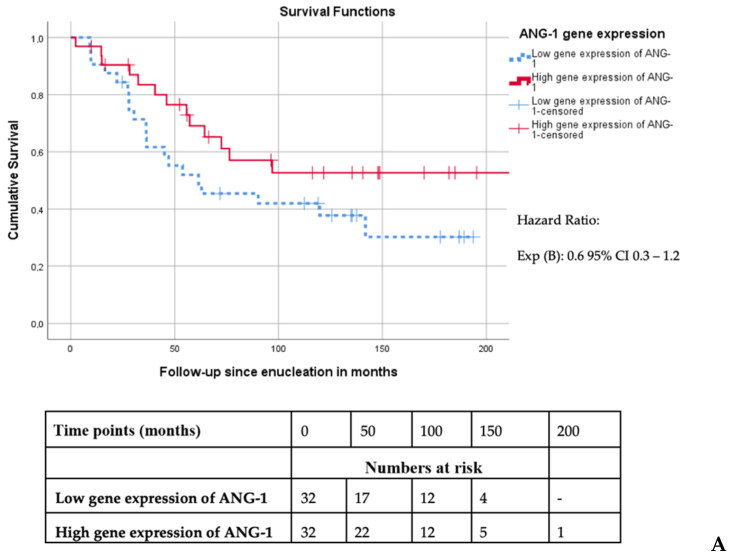

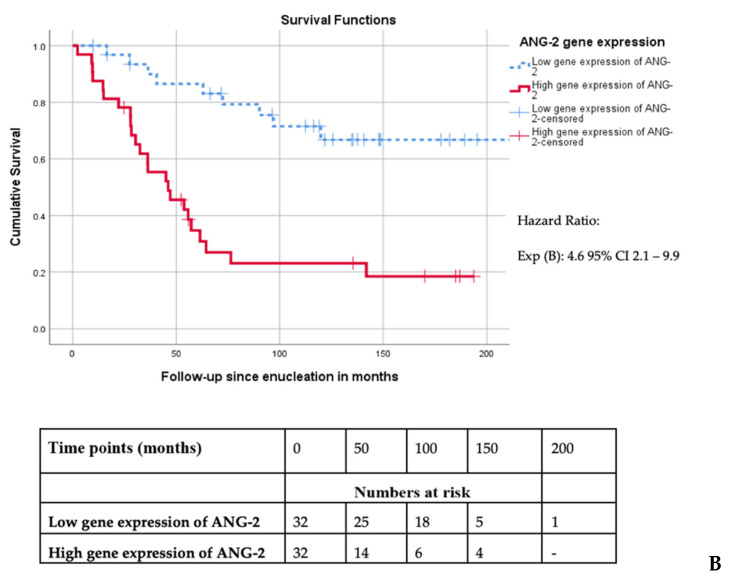
(**A**) Survival curve of UM-related survival dichotomized according to the median mRNA expression of ANG-1 in primary uveal melanoma (*n* = 64, Log Rank *p* = 0.12). (**B**) Survival curve of UM-related survival dichotomized according to the median mRNA expression of ANG-2 in primary uveal melanoma (*n* = 64, Log Rank *p* < 0.001).

When comparing Kaplan–Meier survival curves, we did not find an association between ANG-1 gene expression and disease-specific survival (mean survival of 139 months (high gene expression) versus 97 months (low gene expression), *p* = 0.1) (Figure 1A). However, the group of patients with a high ANG-2 mRNA expression in the tumor had a worse UM-related survival compared to the group with a low ANG-2 gene expression (mean survival 65 versus 169 months, *p* = 0.001) (Figure 1B).

### 3.2. Presence of ANG-1 and ANG-2 Protein in Aqueous Humor

Paracentesis of aqueous humor allows easy access and we recently observed that ANG-1 and ANG-2 can be present in the aqueous humor of a UM-containing eye [42]. Therefore, we set out to analyze whether the concentration of ANG-1 and ANG-2 in aqueous humor is higher in high-risk cases. Concentrations were determined by a multiplex proximity extension assay in 83 aqueous humor samples from eyes enucleated for UM. The median age of the patients at enucleation was 60.0 years, and 54% of the patients were men. The concentration of ANG-1 and ANG-2 was dichotomized, using the median as a cut-off point (the median concentration of ANG-1 protein was 1.31 NPX (range 0.45–4.29 NPX), the median concentration of ANG-2 was 0.73 NPX (range −0.71–8.39 NPX).

Clinical and histopathologic features were compared between the low and high ANG-1 and ANG-2 group, and no association with ANG-1 was observed. A high concentration of ANG-2 in aqueous humor, on the other hand, was associated with a higher age (with a median of 68 years versus 55 years, *p* = 0.002), a larger basal diameter (with a median of 13 versus 12 mm, *p* = 0.04), a greater prominence (with a median of 9 mm versus 7 mm, *p* < 0.001), involvement of the ciliary body (51% versus 19%, *p* = 0.002), and advanced TNM8-stage III (54% stages IIIA-IIIC versus 17%, *p* < 0.001) (Table 2). 

**Figure 2:**
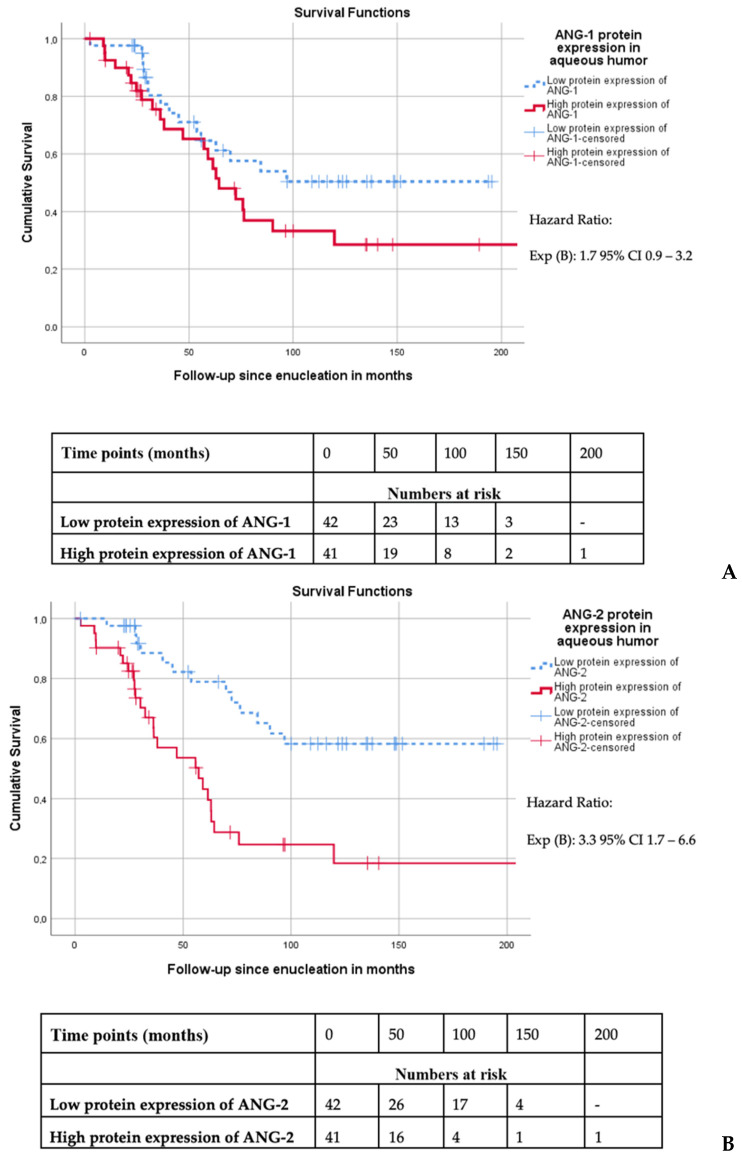
(**A**) Survival curve of UM-related survival dichotomized according to the median of ANG-1 expression levels in the aqueous humor (*n* = 83, Log Rank *p* = 0.11). (**B**) Survival curve of UM-related survival dichotomized according to the median of ANG-2 expression levels in the aqueous humor (*n* = 83, Log Rank *p* < 0.001).

When comparing the Kaplan–Meier survival curves, no significant association between ANG-1 protein expression and disease-specific survival was observed (the low group had a mean survival of 121 months, versus 100 months in the high ANG-1 group, *p* = 0.08) (Figure 2A). In contrast, the high ANG-2 group had a significantly worse disease-specific survival compared to patients in the low ANG-2 group (mean disease-specific survival of 76 versus 142 months, *p* = 0.002) (Figure 2B).

### 3.3. Correlation of Protein and Gene Expression

**Figure 3:**
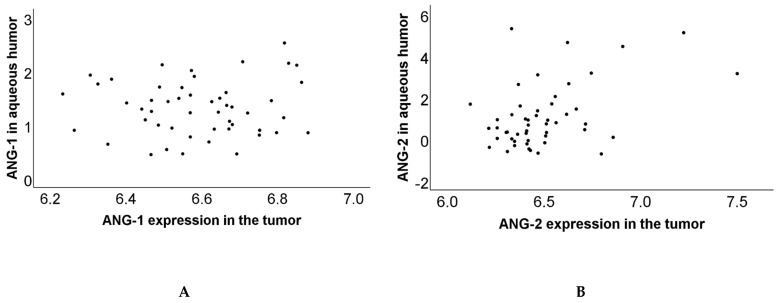
Two scatterplots to visualize the correlation between protein and gene expression of angiopoietins: (**A**) Correlation of ANG-1 protein and mRNA expression (rS = 0.02, *p* = 0.89). (**B**) Correlation of ANG-2 protein and mRNA expression (rS = 0.31, *p* = 0.03).

We subsequently determined whether the concentrations of ANG-1 and ANG-2 in the aqueous humor resembled the gene expression in the tumor tissue. To make both groups comparable, we transformed the protein expression data, which were originally provided as normalized data on a log2 scale, back to a linear scale. A Spearman rank-order correlation coefficient was determined in a group of 50 patients on whom we had data on aqueous humor protein levels as well as on tumor-specific gene expression. The analysis of a correlation between protein and gene expression of both angiopoietins showed no correlation between ANG-1 protein expression in the aqueous humor and ANG-1 mRNA expression in tumor material (rS = 0.02, *p* = 0.89) (Figure 3A), but it did show a positive correlation between ANG-2 protein expression and mRNA expression (rS = 0.31, *p* = 0.03) (Figure 3B).

### 3.4. Association between ANG-2 and Inflammation

**Figure 4:**
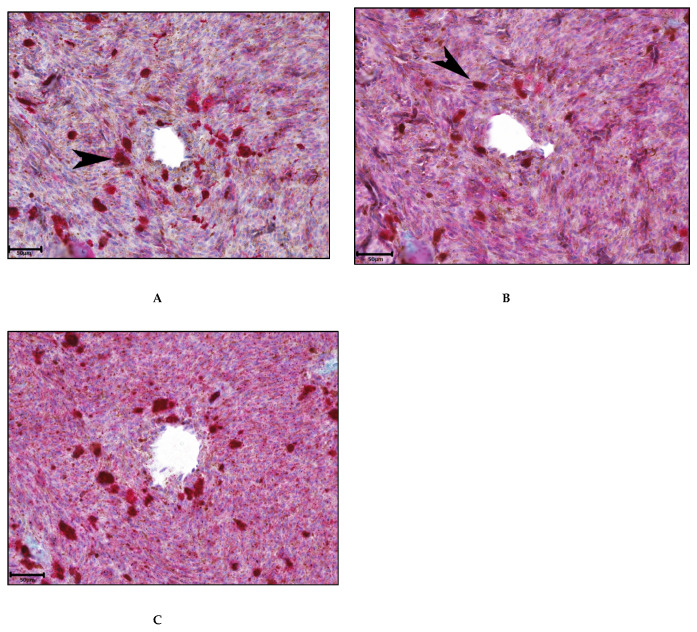
Representative immunohistochemical analysis to determine the expression of ANG-2 and infiltration of T-cells and M2 macrophages in primary tumor material: Scale bar is 50 um. (**A**) CD163 expression to identify M2 macrophages (black arrow); (**B**) CD3 to identify lymphocytes (black arrow); (**C**) ANG-2 expression (original magnification 10.0×, red chromogen).

IHC was performed on ten cases of UM in order to investigate which cells express ANG-2 and to investigate the association between ANG-2 and inflammation. While macrophages and T cells often highly express ANG-2, it is also expressed in tumor cells (Figure 4). No correlation was found between the level of ANG-2 expression and the level of infiltrate in the tumor (data not shown). In the ten cases, there was no significant difference in the percentage of macrophages or CD3+ T cells that express ANG-2. Moreover, the ANG-2-expressing macrophages and T cells were equally distributed among the immune infiltrate in the tumor. Furthermore, we tried to determine which cell type expressed ANG-2 by looking at single cell RNA data from eight primary UM (GSE139829) [43,44]. However, ANG-2 was expressed in a very low fraction (0–3%) of the cells per tumor, which was too marginal to draw any definite conclusions regarding the origin of ANG-2.

## 4. Discussion

The current study showed a significant association between a decreased UM-related survival and a high ANG-2 protein- and mRNA expression in aqueous humor and tumor material. After correcting for multiple individual prognostic parameters, a high ANG-2 protein and a high mRNA gene expression carried a significant hazard ratio (HR) of 3.22 and 0.28, respectively. ANG-2 gene expression was associated with an elderly age, a large tumor basal diameter, the presence of epithelioid cells, a high mitotic count, the presence of monosomy 3, and the occurrence of metastases. As is known from previous studies, the presence of blood vessels is of great importance for the development of metastases in UM [7]. A few proangiogenic factors have already been associated with the increased malignancy of UM, including infiltration by M2 macrophages, and expression of VEGF-A and HGF [10,11,20]. The current study shows that ANG-2 is also associated with decreased UM-related survival, which is in line with previous studies in which the role of angiopoietins has been investigated in other types of cancer, especially in hepatocellular carcinoma and lung cancer [30,34,35,36]. Our findings confirm the important role of ANG-2 in tumor malignancy for UM. Based on the vascular stabilizing effects of ANG-2 inhibition on delaying tumor progression and enhancing immune responses, ANG-2 could be a potential target for (combination) therapy in primary and perhaps metastatic UM. Currently, there are several ongoing clinical trials which test combination therapies with multiple drugs targeting the ANG2/TIE2 pathway. These trials include patients with (metastatic) melanoma, glioma, ovarian cancer, renal cell carcinoma, glioblastoma, metastatic colorectal cancer, and other solid tumors [45,46]. Naturally, the complex function of ANG-2 in the TIE-2 pathway and its interplay with ANG-1 requires more research for the development of drugs against UM.

A study on ANG-2 in human gliomas found overexpression of ANG-2 in the invasive areas of the brain, accompanied by matrix metalloprotease-2 (MMP-2) and increased angiogenesis. Recombinant ANG-2 in glioma cell lines activated MMP-2 expression in tumor cells and promoted invasiveness, leading to the conclusion that ANG-2 plays a critical role in tumor cell infiltration by activating MMP-2 [47]. A study on hepatocellular carcinoma (HCC) found that ANG-2-expressing cells promoted the rapid development of human HCC and produced hemorrhages within tumors in mice, suggesting a role for ANG-2 in neovascularization [48].

It is known from previous studies that, next to proangiogenic factors, an inflammatory phenotype in UM is associated with decreased survival [17,19]. As ANG-2 levels are also associated with a poor prognosis, we wondered whether inflammatory cell infiltration may be the source of ANG-2. We performed IHC staining on ten UM samples and indeed observed expression in M2 macrophages and T cells, but also in tumor cells.

We specifically observed an association between increased levels of ANG-2 in aqueous humor and tumor tissue with the chromosome status of the tumor, and therefore consider ANG-2 as a marker of tumor progression. ANG-2 has been identified as a disturber in the stabilization of developing vessels and promotes proinflammatory and proangiogenic cytokine production and vascular leakage [35,36]. This identifies ANG-2 as an interesting target for therapeutic intervention. The impact of blocking ANG-2 should first be explored in animal models of UM to determine whether this could lead to an effective therapy, especially for (the prevention of) metastatic disease. Recent studies have shown that inhibition of ANG-2 may be used as treatment of diabetic retinopathy and age-related macular degeneration [49]. It would be interesting to determine the presence of this cytokine post-irradiation and consider it a target in radiation retinopathy.

A limitation of the current study is that we used a predetermined cut-off value for the protein and gene expression of ANG-1 and ANG-2 by using the median to discriminate patients with high and low protein and gene expression. As these measurements are not standardized, the used cut-off values of protein and gene expression may not be suitable for other analytic methods. In addition, this study only investigated tumors which were primary enucleated, and these tumors tend to be the large ones, as primary enucleation is, in general, the first choice of treatment for tumors which are too large for radiotherapy. These results require verification in smaller tumors to determine their applicability.

## 5. Conclusions

This study found a positive association between the high expression of ANG-2 (but not of ANG-1) and high-risk primary UM. ANG-2 gene expression was associated with the development of metastases and the presence of monosomy 3. Our findings make ANG-2 an attractive target for the potential treatment of UM.

## Figures and Tables

**Table 1:** Comparison of clinical, histopathological, and genetic characteristics of UM with a low or high mRNA gene expression of ANG-1 and ANG-2. The groups were separated according to the median.

Variables	Patients, No. (%) *		*p*-Value	Patients, No. (%) *		*p*-Value
	Low Gene Expression of ANG-1 (*n* = 32)	High Gene Expression of ANG-1 (*n* = 32)		Low Gene Expression of ANG-2 (*n* = 32)	High Gene Expression of ANG-2 (*n* = 32)	
Sex			0.32 ^‡^			0.32 ^‡^
Male	14 (44%)	18 (56%)		18 (56%)	14 (44%)	
Female	18 (56%)	14 (44%)		14 (44%)	18 (56%)	
Age at enucleation, median (range), years	57.8 (33.4–88.4)	65.9 (12.8–84.8)	0.36 ^§^	56.4 (12.8–84.8)	69.1 (33.4–88.4)	0.04 ^§^
Largest basal diameter, median (range), mm	14.0 (9.0–20.0)	13.0 (8.0–30.0)	0.18 ^§^	12.0 (8.0–18.0)	15.0 (9.0–30.0)	0.01 ^§^
Thickness, median (range), mm	8.5 (2.0–12.0)	7.5 (2.0–12.0)	0.24 ^§^	7.0 (2.0–12.0)	8.5 (4.0–12.0)	0.09 ^§^
Ciliary body involvement			0.002 ^‡^			0.12 ^‡^
No	34 (81%)	20 (49%)		23 (72%)	17 (53%)	
Yes	8 (19%)	21 (51%)		9 (28%)	15 (47%)	
Cell type			0.08 ^‡^			0.04 ^‡^
Spindle	18 (43%)	10 (24%)		15 (47%)	7 (22%)	
Mixed or epithelioid	24 (57%)	31 (76%)		17 (53%)	25 (78%)	
AJCC staging			0.001 ^‡^			0.02 ^‡^
I-IIB	35 (83%)	19 (46%)		25 (78%)	16 (50%)	
IIIA-IIIC	7 (17%)	22 (54%)		7 (22%)	16 (50%)	
Metastases			0.21 ^‡^			0.001 ^‡^
No	11 (34%)	16 (50%)		20 (63%)	7 (22%)	
Yes	22 (66%)	16 (50%)		12 (38%)	25 (78%)	
Chromosome 3 status			0.19 ^‡^			0.004 ^‡^
Disomy 3	9 (28%)	14 (44%)		17 (53%)	6 (19%)	
Monosomy 3	23 (72%)	18 (56%)		15 (47%)	26 (81%)	
Chromosome 8 status			0.78 ^‡^			0.06 ^‡^
Normal	9 (28%)	10 (31%)		13 (41%)	6 (19%)	
Gain of 8q	23 (72%)	22 (69%)		19 (59%)	26 (81%)	

* Percentages are rounded and may therefore not total 100, ‡ Pearson’s chi-squared test, § Mann–Whitney U test.

**Table 2:** Comparison of clinical, histopathological, and genetic characteristics of UM with a low or high protein expression of ANG-1 and ANG-2 in the aqueous. The groups were separated according to the median.

Variables	Patients, No. (%) *		*p*-Value	Patients, No. (%) *		*p*-Value
	Low Protein Expression of ANG-1 (*n* = 42)	High Protein Expression of ANG-1 (*n* = 41)		Low Protein Expression of ANG-2 (*n* = 42)	High Protein Expression of ANG-2 (*n* = 41)	
Sex			0.51 ^‡^			0.92 ^‡^
Male	21 (50%)	24 (59%)		23 (55%)	22 (54%)	
Female	21 (50%)	17 (42%)		19 (45%)	19 (46%)	
Age at enucleation, median (range), years	59.8 (12.8–77.1)	62.3 (32.2–92.7)	0.43 ^§^	54.6 (12.8–77.1)	68.3 (34.8–92.7)	0.002 ^§^
Largest basal diameter, median (range), mm	12.0 (8.0–20.0)	13.0 (9.0–20.0)	0.10 ^§^	12.0 (8.0–18.0)	13.0 (9.0–20.0)	0.04 ^§^
Thickness, median (range), mm	7.0 (2.0–12.0)	9.0 (2.0–12.0)	0.08 ^§^	7.0 (2.0–12.0)	9.0 (2.0–12.0)	< 0.001 ^§^
Ciliary body involvement			0.50 ^‡^			0.002 ^‡^
No	29 (69%)	25 (61%)		34 (81%)	20 (49%)	
Yes	13 (31%)	16 (39%)		8 (19%)	21 (51%)	
Cell type			0.70 ^‡^			0.08 ^‡^
Spindle	15 (36%)	13 (32%)		18 (43%)	10 (24%)	
Mixed or epithelioid	27 (65%)	28 (68%)		24 (57%)	31 (76%)	
AJCC staging			0.76 ^‡^			<0.001 ^‡^
I-IIB	28 (67%)	26 (63%)		35 (83%)	19 (46%)	
IIIA-IIIC	14 (33%)	15 (37%)		7 (17%)	22 (54%)	
Metastases			0.23 ^‡^			0.04 ^‡^
No	24 (57%)	19 (46%)		26 (62%)	16 (39%)	
Yes	18 (43%)	22 (54%)		16 (38%)	25 (62%)	
Chromosome 3 status			0.21 ^‡^			0.42 ^‡^
Disomy 3	20 (48%)	14 (34%)		19 (45%)	15 (37%)	
Monosomy 3	21 (50%)	26 (63%)		22 (52%)	25 (61%)	
Failed analyses	1 (2%)	1 (2%)		1 (2%)	1 (2%)	
Chromosome 8 status			0.68 ^‡^			0.37 ^‡^
Normal	13 (31%)	11 (27%)		14 (33%)	10 (24%)	
Gain of 8q	28 (67%)	29 (71%)		27 (64%)	30 (73%)	
Failed analyses	1 (2%)	1 (2%)		1 (2%)	1 (2%)	

* Percentages are rounded and may therefore not total 100, ‡ Pearson’s chi-squared test, § Mann–Whitney U test.

## Data Availability

Part of the data are available at GEO dataset GSE84976) [40], and additional data can be provided by the authors on request.
